# Comprehensive Evaluation of a Point-of-Care Testing Platform for Decentralized Primary Healthcare: Ensuring Analytical Quality Through Central Laboratory Oversight

**DOI:** 10.3390/diagnostics15232977

**Published:** 2025-11-24

**Authors:** Giacomo Moretti, Francesca Danila Alcaro, Luigi Colacicco, Andrea Urbani

**Affiliations:** 1 Clinical Chemistry, Biochemistry and Molecular Biology Operations (UOC), Fondazione Policlinico Universitario A. Gemelli IRCCS, 00168 Rome, Italy; luigi.colacicco@policlinicogemelli.it (L.C.); andrea.urbani@policlinicogemelli.it (A.U.); 2 Bio-Bank Facility, Fondazione Policlinico Universitario A. Gemelli IRCCS, 00168 Rome, Italy; francescadanila.alcaro@guest.policlinicogemelli.it; 3 Department of Basic Biotechnological Sciences, Intensive Care and Perioperative Clinics Research, Catholic University of the Sacred Heart, 00168 Rome, Italy

**Keywords:** point-of-care testing (POCT), analytical quality, laboratory oversight, method comparison, primary healthcare, decentralized testing, clinical governance, method validation

## Abstract

**Background/Objectives:** Point-of-care testing (POCT) is increasingly adopted in primary healthcare to facilitate rapid screening and monitoring of chronic conditions. Ensuring that its analytical quality is comparable to central laboratory testing is crucial for safe and effective implementation. This study aims to rigorously evaluate the analytical performance of the Allegro POCT system against established central laboratory reference methods to determine its suitability for decentralized healthcare settings. **Methods:** We assessed the correlation, concordance, and bias of glycated hemoglobin (HbA1c), glucose (GLUC), total cholesterol (CHOL), high-density lipoprotein cholesterol (HDL), triglycerides (TRIG), creatinine (CREA), and C-reactive protein (CRP). Using a cohort of 100 residual patient samples, measurements from the Allegro POCT system were compared against reference methods on the Atellica CH 930 Analyzer and TOSOH G8 system. The statistical analysis was performed using Deming regression, Bland–Altman plots, and Pearson correlation. **Results:** HbA1c and GLUC demonstrated strong linearity and correlation (Pearson’s r = 0.9863 and r = 0.9994, respectively). A slight positive bias was noted for HbA1c at higher concentrations. In the lipid panel, CHOL showed a significant positive bias (mean bias +14.2 mg/dL), while TRIG exhibited a substantial negative bias (mean bias −37.0 mg/dL) and wide limits of agreement. HDL and CREA showed good linearity but only moderate agreement. CRP demonstrated excellent concordance with the reference method (Pearson’s r = 0.9955) and minimal bias. **Conclusions:** The Allegro system exhibits acceptable analytical performance for GLUC and CRP, rendering it suitable for decentralized use. HbA1c and CREA performance is adequate, though caution is advised due to observed biases. However, the significant biases for CHOL and TRIG underscore the indispensable role of central laboratory oversight in any POCT program. Rigorous initial validation and continuous quality monitoring under a robust governance framework are essential to ensure the reliability and clinical utility of POCT.

## 1. Introduction

Point-of-care testing (POCT) has emerged as a transformative approach in healthcare, bringing laboratory diagnostics closer to the patient to facilitate rapid clinical decision-making. Designed for simplicity and ease of use, POCT devices enable healthcare professionals with basic training to process whole blood samples and obtain timely results, circumventing the delays often associated with central laboratory testing. The advantages of POCT are well-documented and include reduced turnaround time (TAT), improved patient outcomes, decreased length of hospital stay, and enhanced cost-effectiveness [1, 2, 3, 4, 5]. These benefits make POCT particularly valuable in primary care settings, where rapid screening and therapeutic monitoring can significantly impact patient management.

The global COVID-19 pandemic further accelerated the adoption of POCT, as laboratories worldwide sought to decentralize testing and support patient care outside of traditional hospital environments. This surge in POCT utilization highlighted its value in areas with limited access to laboratory infrastructure or trained personnel, reinforcing its expanding role in primary healthcare [6, 7, 8, 9, 10].

Despite its increasing popularity, the analytical performance of POCT devices has historically been a subject of scrutiny. Earlier generations of POCT systems often lacked the accuracy and precision of central laboratory instruments, leading to justifiable hesitation regarding their widespread adoption. However, recent technological advancements have substantially improved the analytical concordance between many POCT platforms and laboratory-based methods [2, 5, 11, 12, 13, 14]. This is particularly true for modern multi-analyte platforms like the Allegro system, which consolidate different detection principles—such as latex agglutination immunoassays, enzymatic colorimetric assays, and amperometric biosensors—into a single device. The contribution of our study is to evaluate such a consolidated system not as a standalone device, but as a component within an integrated healthcare model. This progress has fostered greater confidence in POCT and facilitated its increasing implementation, particularly in primary care settings.

A non-negotiable aspect of successful POCT implementation is ensuring the quality and reliability of results. International standards, including ISO 15189 [15] and ISO 22870 [16], explicitly emphasize the importance of rigorous quality control procedures. These include consistent internal quality control (IQC) and participation in external quality assessment (EQA) schemes for all testing modalities, POCT included. The seamless integration of POCT data with hospital information systems under the direct supervision, validation, and quality oversight of the central laboratory is crucial for ensuring data integrity, traceability, and clinical governance [11, 17].

In line with the expanding model of a “laboratory spread throughout the territory,” conceptualized as a “hub-and-spoke” model of clinical governance where the central laboratory (the hub) provides direct oversight, quality assurance, and data integration for multiple decentralized testing points (the spokes), our institution recognized the critical need to thoroughly evaluate POCT platforms prior to their deployment. This study was undertaken to comprehensively assess the analytical performance of the Allegro POCT system (Nova Biomedical Italia S.r.l.^®^, Lainate (MI), Italy) in direct comparison with the established reference methods employed in our central laboratory. The primary objective was not only to determine the analytical suitability of the Allegro for basic screening and monitoring but also to establish a validation framework and define the operational requirements for its safe and effective deployment within a centrally governed primary care network.

## 2. Materials and Methods

### 2.1. Study Design and Ethical Considerations

This analytical method comparison study was conducted using a cohort of 100 residual patient specimens obtained from the routine workflow of the “Agostino Gemelli” IRCCS University Hospital in Rome, Italy. The study protocol received ethical approval from the Institutional Review Board (protocol no. 0002596/22), and all procedures were performed in accordance with the ethical standards of the Declaration of Helsinki. To ensure a robust evaluation, samples were specifically selected from the residual routine workflow to cover a broad and clinically relevant measurement range for each analyte, thus ensuring the robustness of the method comparison across sub-therapeutic, therapeutic, and pathological concentrations. As the study utilized de-identified residual samples that would have otherwise been discarded after routine clinical analysis, specific informed consent for this study was waived. The use of these samples had no impact on patient care or diagnosis.

### 2.2. Sample Collection and Handling

For the determination of glycated hemoglobin (HbA1c), whole blood samples were collected in K3EDTA vacuum tubes (Greiner Bio-One, Kremsmünster, Austria). For all other analytes, including glucose (GLUC), total cholesterol (CHOL), high-density lipoprotein cholesterol (HDL), triglycerides (TRIG), creatinine (CREA), and C-reactive protein (CRP), samples were collected in lithium heparin (LH) vacuum tubes (Greiner Bio-One). To minimize pre-analytical variability, stringent protocols were followed. Particular attention was given to the prompt processing of samples, especially for glucose analysis in lithium heparin tubes which lack a glycolysis inhibitor, to ensure sample integrity and accuracy. All samples were analyzed on the POCT system immediately following their routine assessment on the central laboratory instruments.

### 2.3. Point-of-Care Testing System and Procedures

The Allegro POCT system (Nova Biomedical Italia S.r.l.^®^, Lainate, Milan, Italy), which comprises the Allegro Analyzer (ALL-AN) and the StatStrip A Meter Analyzer (STAST-AN), was utilized for all POCT analyses. For the diabetic profile, HbA1c was determined on the ALL-AN using a latex agglutination inhibition immunoassay, while glucose was measured on the STAST-AN using a disposable test strip employing an amperometric method. The lipid profile, including CHOL, HDL, and TRIG, was analyzed on the ALL-AN through a combination of immunometric, enzymatic, and colorimetric techniques that result in the generation of a blue-colored complex detected by a spectrophotometer. For the kidney profile, creatinine was quantified on the STAST-AN using a test strip-based amperometric method analogous to that for glucose. Lastly, the inflammatory profile marker CRP was assayed on the ALL-AN using a latex particle-enhanced immunoturbidimetric method. All procedures were performed according to the manufacturer’s instructions.

### 2.4. Central Laboratory Reference Methods

To provide a robust and reliable benchmark for evaluation, established reference methods routinely employed in our ISO 15189-accredited central laboratory were used. HbA1c was quantified on a TOSOH G8 analyzer (Tosoh Bioscience^®^ S.r.l., Turin, Italy) using high-performance liquid chromatography (HPLC). Analyses for GLUC, CHOL, HDL, TRIG, and CREA were performed on an Atellica CH 930 Analyzer (Siemens Healthcare^®^ s.r.l., Milan, Italy), using standardized enzymatic methods on lithium-heparin plasma. CRP was also measured on the Atellica platform using an immunoturbidimetric method on plasma. The instrumentation and procedures within the central laboratory are subject to stringent quality control measures, ensuring analytical accuracy and reliability. Manufacturer-provided specifications for all assessed methods are detailed in Table 1.

### 2.5. Statistical Analysis

Statistical analysis was performed using MedCalc software (v20.0, MedCalc Software Ltd., Ostend, Belgium). The comparison between the POCT and central laboratory methods was evaluated using Deming linear regression, which accounts for potential analytical errors in both methods. The strength of the linear association was quantified using Pearson’s correlation coefficient (r). Method agreement, including systematic bias and 95% limits of agreement, was assessed and visualized using Bland–Altman analysis. Prior to statistical analysis, all data were visually inspected for normality using scatter plots and histograms. Outliers were identified using the Bland–Altman plot method (points falling outside ±3 standard deviations from the mean difference) and were investigated for potential pre-analytical or analytical errors. No data points were excluded from the final analysis, as none were attributable to identifiable errors. For the interpretation of the results, a strong linear correlation was pre-defined as a Pearson’s r > 0.95. The 95% confidence intervals for the slope and intercept of the Deming regression were calculated to assess the presence of significant proportional and constant bias, respectively.

## 3. Results

The analytical performance of the Allegro POCT system was evaluated against central laboratory reference methods for seven analytes. A comprehensive summary of the statistical results from Deming regression and Bland–Altman analyses is presented in Table 2.

### 3.1. Diabetic Profile (HbA1c and GLUC)

**Figure 1:**
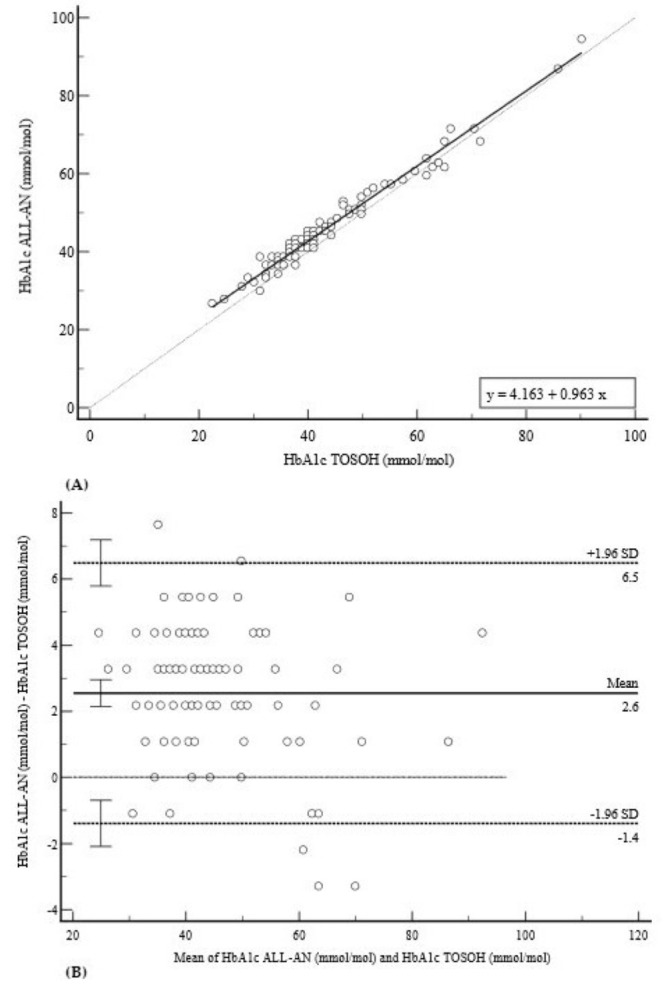
Comparison of methods for HbA1c measurement. (**A**) Deming regression analysis comparing HbA1c measurements (mmol/mol). The solid line represents the Deming regression (y = 4.163 + 0.963x); the dashed line is the line of identity (y = x). (**B**) Bland–Altman plot of the difference against the mean of the two methods. The solid line indicates a mean positive bias of +2.6 mmol/mol, with 95% limits of agreement from −1.4 to 6.5 mmol/mol (dashed lines). Notably, the plot shows a clear trend toward a greater positive difference at higher HbA1c concentrations, confirming the presence of a proportional bias.

The comparison for HbA1c, depicted in Figure 1, showed a strong correlation (Pearson’s r = 0.9863) between the ALL-AN and the TOSOH G8. Deming regression yielded the equation y = 4.1630 + 0.9629x (Figure 1A). The Bland–Altman plot (Figure 1B) indicated a mean positive bias of +2.6 mmol/mol (95% CI: 2.14 to 2.96), with 95% limits of agreement from −1.4 to 6.5 mmol/mol, revealing a tendency toward overestimation by the POCT device, particularly at higher concentrations.

**Figure 2:**
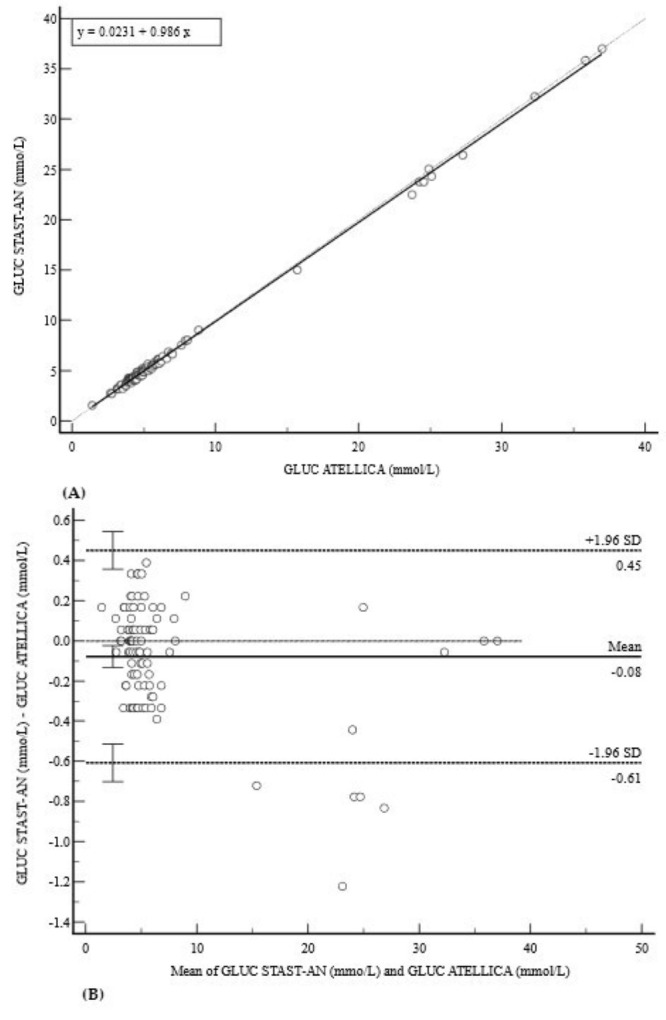
Comparison of methods for GLUC measurement. (**A**) Deming regression analysis comparing GLUC measurements (mmol/L). The solid line represents the Deming regression (y = 0.023 + 0.986x); the dashed line is the line of identity (y = x). (**B**) Bland–Altman plot showing the difference against the mean. The minimal mean bias of −0.08 mmol/L and narrow 95% limits of agreement (−0.61 to 0.45 mmol/L) demonstrate excellent concordance. The data points are randomly scattered around the mean bias, indicating the absence of proportional bias across the measurement range.

For GLUC, the STAST-AN demonstrated excellent agreement with the Atellica reference method (Figure 2). The regression equation was y = 0.02314 + 0.9858x, with a nearly perfect correlation (r = 0.9994) (Figure 2A). This was confirmed by a minimal mean bias of −0.08 mmol/L (95% CI: −0.13 to −0.02) and very narrow limits of agreement (−0.61 to 0.45 mmol/L) (Figure 2B).

### 3.2. Lipid Profile (CHOL, HDL, and TRIG)

**Figure 3:**
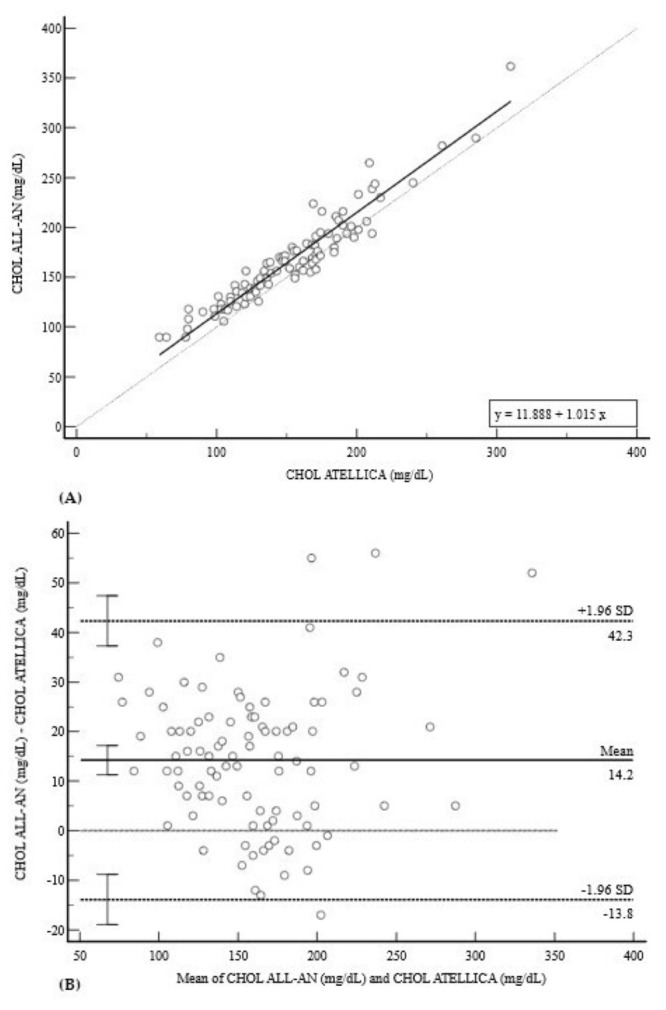
Comparison of methods for CHOL measurement. (**A**) Deming regression analysis for CHOL measurements (mg/dL). The solid line represents the Deming regression (y = 11.888 + 1.015x); the dashed line is the line of identity (y = x). (**B**) Bland–Altman plot revealing a substantial positive mean bias of +14.2 mg/dL, with wide 95% limits of agreement (−13.8 to 42.3 mg/dL). A slight positive trend in the differences at higher concentrations suggests a minor proportional overestimation by the POCT device in addition to the significant constant bias.

The CHOL assay on the ALL-AN exhibited a notable overestimation (Figure 3). Deming regression yielded an equation of y = 11.8882 + 1.0154x (r = 0.9510) (Figure 3A). The Bland–Altman analysis revealed a substantial positive mean bias of +14.2 mg/dL (95% CI: 11.3 to 17.2) with wide limits of agreement (−13.8 to 42.3 mg/dL) (Figure 3B).

**Figure 4:**
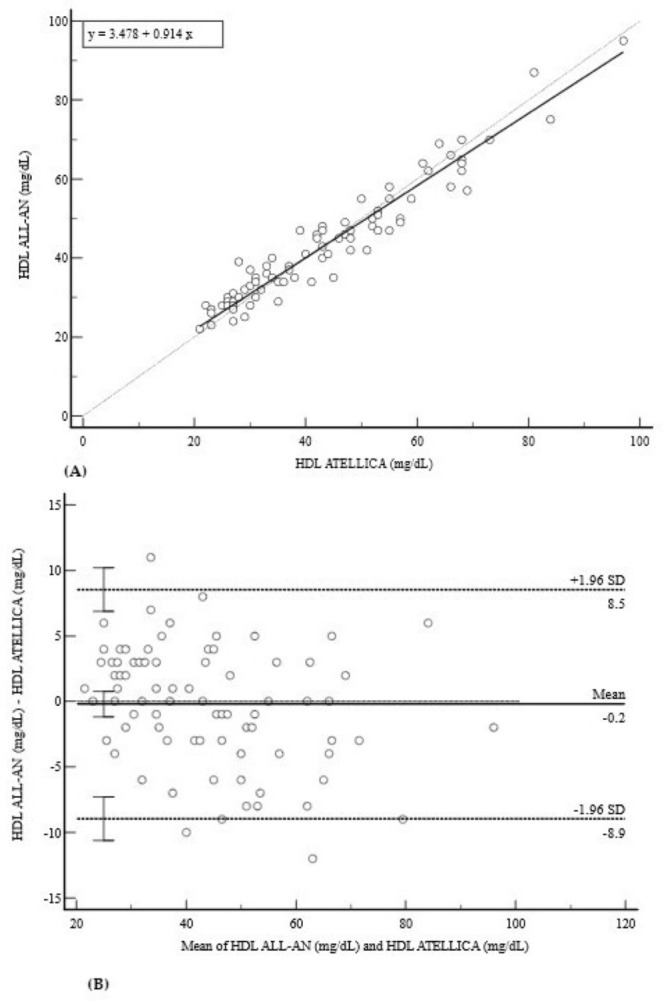
Comparison of methods for HDL measurement. (**A**) Deming regression analysis for HDL measurements (mg/dL). The solid line is the Deming regression (y = 3.478 + 0.914x); the dashed line is the line of identity (y = x). (**B**) Bland–Altman plot indicating a clinically negligible mean bias of −0.2 mg/dL. The 95% limits of agreement are moderately wide (−8.9 to 8.5 mg/dL). The scatter of data points is random, suggesting no significant proportional bias.

For HDL, the results indicated good linearity (y = 3.4780 + 0.9143x; r = 0.9640), as shown in Figure 4A. The mean bias was clinically negligible at −0.2 mg/dL (95% CI: −1.17 to 0.77), although the limits of agreement were moderately wide (−8.9 to 8.5 mg/dL) (Figure 4B).

**Figure 5:**
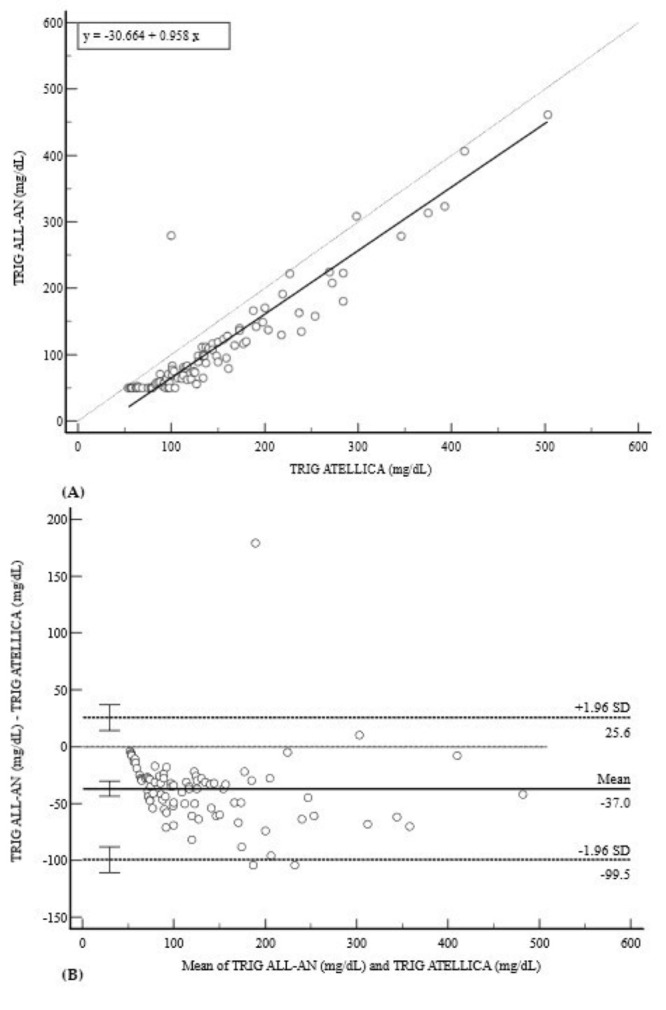
Comparison of methods for TRIG measurement. (**A**) Deming regression analysis for TRIG measurements (mg/dL). The solid line represents the Deming regression (y = −30.664 + 0.958x); the dashed line is the line of identity (y = x). (**B**) Bland–Altman plot demonstrating a large negative mean bias of −37.0 mg/dL with remarkably wide 95% limits of agreement (−99.5 to 25.6 mg/dL). A pronounced negative trend is visible, indicating that the underestimation by the POCT system becomes significantly larger at higher triglyceride concentrations, which confirms a strong proportional bias.

Conversely, a significant underestimation was observed for TRIG (Figure 5). The regression analysis showed weaker correlation (r = 0.9287) with the equation y = −30.6643 + 0.9576x (Figure 5A). This was supported by a large negative mean bias of −37.0 mg/dL (95% CI: −43.6 to −30.4) and remarkably wide limits of agreement (−99.5 to 25.6 mg/dL) (Figure 5B).

### 3.3. Kidney Profile (CREA)

**Figure 6:**
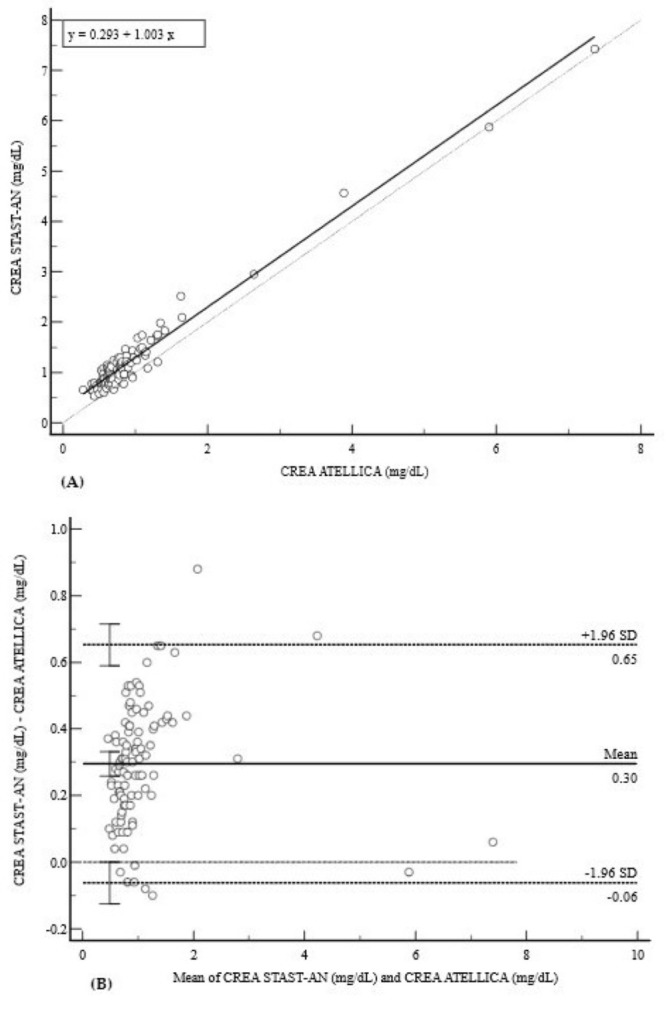
Comparison of methods for CREA measurement. (**A**) Deming regression analysis for CREA measurements (mg/dL). The solid line is the Deming regression (y = 0.293 + 1.003x); the dashed line is the line of identity (y = x). (**B**) Bland–Altman plot showing a slight positive mean bias of +0.30 mg/dL with clinically acceptable 95% limits of agreement (−0.06 to 0.65 mg/dL). The random distribution of data points around the mean bias indicates acceptable performance for monitoring, without evidence of significant proportional bias.

The assessment of CREA on the STAST-AN showed good overall linearity and correlation (y = 0.2926 + 1.0025x; r = 0.9816), as presented in Figure 6A. The Bland–Altman analysis demonstrated a slight positive mean bias of +0.30 mg/dL (95% CI: 0.26 to 0.33), with relatively narrow limits of agreement from −0.06 mg/dL to 0.65 mg/dL, indicating acceptable performance for routine monitoring (Figure 6B).

### 3.4. Inflammatory Profile (CRP)

**Figure 7:**
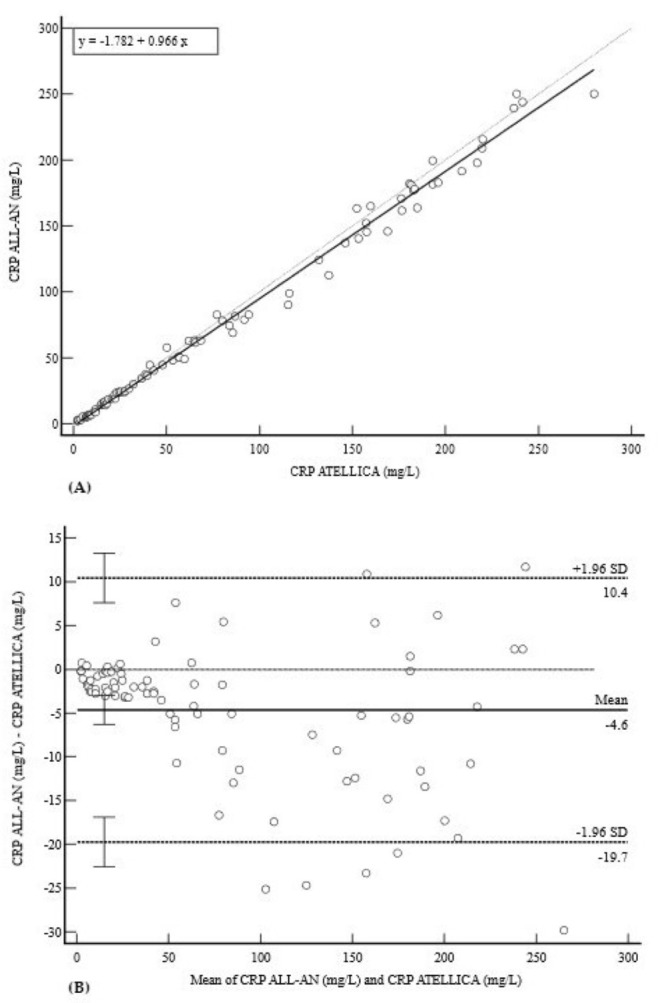
Comparison of methods for CRP measurement. (**A**) Deming regression analysis for CRP measurements (mg/L). The solid line represents the Deming regression (y = −1.782 + 0.966x); the dashed line is the line of identity (y = x). (**B**) Bland–Altman plot showing a minimal mean bias of −4.6 mg/L with wide but clinically acceptable 95% limits of agreement (−19.7 to 10.4 mg/L) for an inflammatory marker. There is no apparent trend in the data, suggesting the method’s bias is consistent across the analytical range.

Finally, the CRP assay on the ALL-AN demonstrated very good agreement with the reference method (Figure 7). The strong correlation (r = 0.9955) was supported by the regression equation y = −1.7818 + 0.9660x (Figure 7A). The Bland–Altman plot showed a minimal mean bias of −4.6 mg/L (95% CI: −6.3 to −3.0) and clinically acceptable limits of agreement from −19.7 mg/L to 10.4 mg/L (Figure 7B).

## 4. Discussion

This study provides a rigorous evaluation of the Allegro POCT system’s analytical performance, an essential prerequisite for its potential deployment in decentralized primary healthcare settings under the governance of a central laboratory. Our findings reveal a complex and nuanced performance profile across different analyte panels, underscoring the necessity for assay-specific assessment and a robust quality management framework.

The results for the diabetic profile were generally encouraging. The exceptional agreement of the GLUC assay confirms its suitability for both screening and therapeutic monitoring in a POCT environment. While the HbA1c assay displayed a slight tendency towards overestimation, particularly at higher concentrations, the magnitude of this bias (mean: 6.3%) might be considered clinically acceptable for screening purposes. However, it mandates careful clinical judgment, and confirmatory testing with a central laboratory method should be standard practice for values near diagnostic thresholds.

The lipid profile presented a more challenging scenario. The substantial positive bias for CHOL (+9.9%) and the large negative bias for TRIG (−33.1%), both accompanied by wide limits of agreement, raise serious concerns regarding their clinical reliability. The origins of these biases are likely multifactorial. One major contributing factor could be the sample matrix effect, as the POCT system utilizes whole blood while the reference method uses plasma. For lipid measurements, physiological variations in hematocrit can significantly influence results from whole blood by altering the plasma volume in the analyzed aliquot. Furthermore, potential interference from erythrocyte components or differences in the enzymatic reagent specificity between the POCT cartridges and the central laboratory assays could contribute to both constant and proportional bias. Lastly, subtle differences in calibration traceability against international reference materials for cholesterol and triglycerides may be amplified in a POCT system designed for broad use. A bias of this magnitude, particularly for TRIG, could lead to significant misclassification of patients’ cardiovascular risk profiles and, consequently, to inappropriate treatment decisions. The HDL assay performed acceptably, but the poor performance of the TRIG assay indicates it is unsuitable for clinical use without significant method recalibration or optimization.

The assays for CREA and CRP yielded more reassuring results. The slight overestimation of CREA is unlikely to be clinically significant in most monitoring scenarios, and its relatively narrow limits of agreement suggest sufficient accuracy. The CRP assay demonstrated excellent agreement, supporting its utility for the rapid assessment of inflammatory status in primary care.

When contextualized with other widespread commercial POCT systems, the Allegro’s performance reveals a competitive but varied profile. For instance, platforms like the Afinion™ Analyzer are well-regarded for their HbA1c performance, often showing excellent agreement with HPLC methods, comparable to what we observed. Conversely, multi-analyte systems like the i-STAT^®^ have demonstrated robust performance for analytes like creatinine and glucose but also exhibit method-specific biases that require clinical awareness. The significant lipid biases we identified for the Allegro appear more pronounced than those reported for some other platforms, reinforcing that analytical performance, especially for metabolically complex analytes like triglycerides, remains a heterogeneous and challenging domain in POCT development [18, 19, 20].

These diverse performance profiles highlight that discrepancies between POCT and central laboratory instruments are often analyte-specific and can arise from multiple factors. Differences in methodology and sample matrix (whole blood for POCT vs. plasma for central laboratory assays) are well-known variables that can lead to analytical biases. This heterogeneity in performance demonstrates that a “one-size-fits-all” approach to POCT implementation is inappropriate and potentially detrimental. Each individual assay requires independent evaluation against established reference methods before clinical deployment.

It is, however, important to acknowledge the limitations of this study, which provide context for our findings and outline future research directions. Firstly, our evaluation was performed using venous whole blood samples rather than capillary specimens obtained via finger-prick. This is a significant consideration, as capillary blood is the intended and most common sample type for many POCT applications. Potential differences in composition between venous and capillary blood, alongside pre-analytical variability inherent in capillary collection, could negatively affect analytical performance. Therefore, the reported agreement may be overestimated compared to real-world clinical use. Secondly, all analyses were conducted by trained laboratory professionals in a controlled setting. This ensures methodological consistency but does not fully replicate the conditions of a typical primary care environment, where testing is often performed by clinical staff with varied levels of technical expertise. The performance of any POCT system can be operator-dependent, and the potential for increased imprecision or error may arise when used by non-laboratory personnel. This underscores the critical importance of implementing standardized training, continuous education, and competency assessment programs governed by the central laboratory as part of any decentralized testing framework. Consequently, future research should prioritize three key areas: (1) a dedicated method comparison using capillary samples to assess performance in a true real-world scenario; (2) an evaluation of long-term analytical stability and lot-to-lot reagent consistency; and (3) usability and performance studies conducted directly by primary care staff to assess the impact of operator variability.

To translate these findings into a practical decentralized primary healthcare framework, several operational factors beyond analytical performance must be considered. A comprehensive cost–benefit analysis is necessary to justify implementation. Crucially, a robust, centrally managed operator training and certification program is non-negotiable to minimize user-related errors. Finally, ensuring seamless digital connectivity of POCT devices with laboratory and electronic health record systems is paramount for real-time quality monitoring, immediate result availability, and robust clinical governance.

Ultimately, this study reinforces the indispensable role of the central laboratory in governing any decentralized testing program. The laboratory’s expertise is paramount for conducting initial method validations, establishing and monitoring robust IQC and EQA schemes, providing comprehensive operator training, and managing data integration. A collaborative model founded on seamless communication between the central laboratory and primary healthcare providers is vital to ensure that POCT effectively fulfills its promise of enhancing patient care while safeguarding the fundamental integrity and reliability of laboratory diagnostics.

## 5. Conclusions

The Allegro POCT system demonstrates considerable potential for expanding patient access to essential laboratory testing in primary healthcare settings. Its analytical performance is excellent for GLUC and CRP and acceptable for CREA and HbA1c, provided clinicians are aware of the inherent analytical biases and use the results accordingly. However, the significant inaccuracies observed for CHOL and, most notably, for TRIG, forcefully emphasize that safe and effective POCT implementation requires more than simple device deployment. It necessitates a comprehensive framework of continuous and vigilant central laboratory oversight, encompassing rigorous initial validation, ongoing quality assurance, and a clear collaborative governance structure. Looking forward, continued methodological optimization by manufacturers and the integration of digital, cloud-based quality-tracking systems will be key to elevating scientific depth and operational control. This study re-emphasizes the crucial role of the central laboratory in clinical translation—specifically, in providing the quality assurance framework required to ensure that the promise of POCT technology can be implemented safely and effectively for tangible patient benefit.

## Figures and Tables

**Table 1:** Manufacturer-provided analytical characteristics of the Allegro POCT system and central laboratory reference methods.

Analyte	System	Method/Principle	Required Volume (µL)	Time to Result	Linearity Range	Reference Range	Matrix
HbA1c	Allegro (Nova Biomedical Italia S.r.l.^®^, Lainate, Milan, Italy, ALL-AN)	Latex Agglutination Immunoassay	1.5	6 min	10–130 mmol/mol	<42 mmol/mol	Whole Blood (K_3_EDTA)
	Central Lab (Tosoh Bioscience^®^ S.r.l., Turin, Italy, TOSOH)	HPLC	4	1.6 min	N/A (calibrated range)	<42 mmol/mol	Whole Blood (K_3_EDTA)
Glucose	Allegro (STAST-AN)	Amperometric Biosensor	1.2	6 s	10–600 mg/dL	65–100 mg/dL	Whole Blood (LH)
	Central Lab (Siemens Healthcare^®^ s.r.l., Milan, Italy, Atellica)	Enzymatic (Hexokinase)	5	10 min	4–700 mg/dL	65–100 mg/dL	Plasma (LH)
Cholesterol	Allegro (ALL-AN)	Enzymatic-Colorimetric	80	10 min	25–620 mg/dL	<200 mg/dL	Whole Blood (LH)
	Central Lab (Atellica)	Enzymatic-Colorimetric	5	10 min	90–500 mg/dL	<200 mg/dL	Plasma (LH)
HDL	Allegro (ALL-AN)	Immunometric	80	10 min	5–129 mg/dL	>45 mg/dL	Whole Blood (LH)
	Central Lab (Atellica)	Enzymatic-Colorimetric (Direct)	5	10 min	20–100 mg/dL	>45 mg/dL	Plasma (LH)
Triglycerides	Allegro (ALL-AN)	Enzymatic-Colorimetric	80	10 min	10–600 mg/dL	<150 mg/dL	Whole Blood (LH)
	Central Lab (Atellica)	Enzymatic-Colorimetric	5	10 min	50–550 mg/dL	<150 mg/dL	Plasma (LH)
Creatinine	Allegro (STAST-AN)	Amperometric Biosensor	1.2	30 s	0.3–12 mg/dL	0.67–1.17 mg/dL	Whole Blood (LH)
	Central Lab (Atellica)	Enzymatic (Creatinase)	5	10 min	0.1–30 mg/dL	0.67–1.17 mg/dL	Plasma (LH)
CRP	Allegro (ALL-AN)	Latex-Enhanced Immunoturbidimetry	5	7 min	2.0–250 mg/L	<5.0 mg/L	Whole Blood (LH)
	Central Lab (Atellica)	Immunoturbidimetry	40	10 min	0.5–156 mg/L	<5.0 mg/L	Plasma (LH)

Abbreviations: POCT, point-of-care testing; ALL-AN, Allegro Analyzer; STAST-AN, StatStrip A Meter Analyzer; HPLC, High-Performance Liquid Chromatography; LH, Lithium Heparin; K_3_EDTA, tripotassium ethylenediaminetetraacetic acid; N/A, not applicable. Central Laboratory platforms were the TOSOH G8 analyzer for HbA1c and the Siemens Atellica CH 930 Analyzer for all other analytes.

**Table 2:** Summary of method comparison results from Deming regression and Bland–Altman analysis.

Analyte (Units)	Pearson’s r (95% CI)	Deming Intercept (95% CI)	Deming Slope (95% CI)	Mean Bias (95% LoA)
HbA1c (mmol/mol)	0.9863 (0.98–0.99)	4.16 (2.50 to 5.82) *	0.96 (0.93 to 0.99) *	+2.6 (−1.4 to 6.5)
GLUC (mmol/L)	0.9994 (0.99–1.00)	0.02 (−0.05 to 0.10)	0.98 (0.97 to 1.00)	−0.08 (−0.61 to 0.45)
CHOL (mg/dL)	0.9510 (0.93–0.97)	11.89 (4.50 to 19.28) *	1.01 (0.98 to 1.05)	+14.2 (−13.8 to 42.3)
HDL (mg/dL)	0.9640 (0.95–0.97)	3.48 (−0.50 to 7.46)	0.91 (0.86 to 0.97)	−0.2 (−8.9 to 8.5)
TRIG (mg/dL)	0.9287 (0.90–0.95)	−30.66 (−45.1 to −16.22) *	0.96 (0.91 to 1.01)	−37.0 (−99.5 to 25.6)
CREA (mg/dL)	0.9816 (0.97–0.99)	0.29 (0.24 to 0.35) *	1.00 (0.95 to 1.05)	+0.3 (−0.06 to 0.65)
CRP (mg/L)	0.9955 (0.99–1.00)	−1.78 (−3.5 to 0.0)	0.97 (0.95 to 0.99)	−4.6 (−19.7 to 10.4)

Footnotes: Glycated hemoglobin HbA1c; glucose GLUC; total cholesterol CHOL; high density lipoprotein cholesterol HDL; triglycerides TRIG; creatinine CREA; C-reactive protein CRP; confidence interval CI; limits of agreement LoA. * Indicates a 95% CI for the intercept that does not include the ideal value of 0 or for the slope that does not include the ideal value of 1, suggesting a statistically significant constant or proportional bias, respectively.

## Data Availability

The data presented in this study are available within the article. Further raw data may be made available upon reasonable request to the corresponding author.

## Abbreviations

The following abbreviations are used in this manuscript:

POCT	Point-of-Care Testing
HbA1c	Glycated Hemoglobin
GLUC	Glucose
CHOL	Total Cholesterol
HDL	High-Density Lipoprotein Cholesterol
TRIG	Triglycerides
CREA	Creatinine
CRP	C-Reactive Protein
IQC	Internal Quality Control
EQA	External Quality Assessment
LH	Lithium Heparin
ALL-AN	Allegro Analyzer
STAST-AN	StatStrip A Meter Analyzer
HPLC	High-Performance Liquid Chromatography
